# Using a modified Delphi procedure to select a PRO-CTCAE-based subset for patient-reported symptomatic toxicity monitoring in rectal cancer patients

**DOI:** 10.1007/s11136-024-03767-0

**Published:** 2024-09-08

**Authors:** Yvonne M. Geurts, Femke Peters, Esther Feldman, Jeanine Roodhart, Milan Richir, Jan Willem T. Dekker, Geerard Beets, Jeltsje S. Cnossen, Patricia Bottenberg, Martijn Intven, Marcel Verheij, Kelly M. de Ligt, Iris Walraven

**Affiliations:** 1 Department of IQ Health, Radboud University Medical Center, Postbus 9101, 6500 HB Nijmegen, The Netherlands; 2 Department of Radiation Oncology, The Netherlands Cancer Institute, Amsterdam, The Netherlands; 3 Department of Medical Oncology, University Medical Centre Utrecht, Utrecht, The Netherlands; 4 Department of Surgery, University Medical Centre Utrecht, Utrecht, The Netherlands; 5 Department of Surgery, Reinier de Graaf Groep, Delft, The Netherlands; 6 Department of Surgery, The Netherlands Cancer Institute, Amsterdam, The Netherlands; 7 GROW School for Oncology and Reproduction, Maastricht University, Maastricht, The Netherlands; 8 Department of Radiation Oncology, Catharina Hospital, Eindhoven, The Netherlands; 9 Department of Radiotherapy, University Medical Centre Utrecht, Utrecht, The Netherlands; 10 Department of Radiation Oncology, Radboud University Medical Center, Nijmegen, The Netherlands; 11 Department of Psychosocial Research and Epidemiology, The Netherlands Cancer Institute, Amsterdam, The Netherlands

**Keywords:** Rectal neoplasms, Patient reported outcome measures, Quality of life, Patient-centered care, Mixed methods

## Abstract

**Purpose:**

Standardized patient-reported outcomes (PRO) monitoring during and after rectal cancer treatment provides insight into treatment-related toxicities patients experience and improves health-related quality-of-life as well as overall survival. We aimed to select a subset of the PRO version of the Common Terminology Criteria for Adverse Events (PRO-CTCAE) for standardized monitoring of treatment-related symptomatic toxicities in rectal cancer.

**Methods:**

We used a mixed methods approach including a literature review, and semi-structured interviews with health care providers (HCPs) involved in rectal cancer care and rectal cancer patients. Results from literature and interviews were summarized and used in a modified Delphi procedure to select a PRO-CTCAE subset specific for rectal cancer.

**Results:**

Twenty-six PRO-CTCAE symptomatic toxicities were identified from literature. Fifteen HCPs from multiple disciplines (medical, radiation and surgical oncology), and a heterogeneous group of fifteen rectal cancer patients treated with chemotherapy and/or radiotherapy and/or surgery, participated in semi-structured interviews. Ten HCPs (67%) and nine patients (90%) participated in the first Delphi round. The final selected PRO-CTCAE core-subset contained 16 symptomatic toxicities: ‘diarrhea’, ‘fecal incontinence’, ‘constipation’,‘bloating of the abdomen’, ‘pain in the abdomen’, ‘vomiting’, ‘decreased libido’, ‘pain during vaginal sex’, ‘ability to achieve and maintain erection’, ‘fatigue’, ‘anxiety’, ‘feeling that nothing could cheer you up’, ‘urinary incontinence’, ‘painful urination’, ‘general pain’, and ‘hand-foot syndrome’.

**Conclusion:**

Based on a comprehensive mixed methods study, a PRO-CTCAE subset for standardized treatment-related symptomatic toxicity monitoring in rectal cancer was identified. Assessment of the effectiveness and compliance of symptomatic toxicity monitoring using this subset is recommended.

**Supplementary Information:**

The online version contains supplementary material available at 10.1007/s11136-024-03767-0.

## Plain English summary

Rectal cancer treatment can result in a wide variety of symptoms that have impact on the health-related quality-of-life of patients. Monitoring treatment-related symptoms experienced by patients using a short, standardized questionnaire which patients can fill out themselves can contribute to an earlier response to these symptoms and therefore prevent negative downstream consequences. The purpose of this study was to select a subset of treatment-related symptoms from the Patient-Reported Outcomes version of the Common Terminology Criteria for Adverse Events (PRO-CTCAE) to be monitored during and after treatment of rectal cancer. To select this subset, a literature review, interviews with health care providers involved in rectal cancer care and rectal cancer patients, and a joint a focus group with both health care providers and rectal cancer patients were performed. Using this mixed method approach, we identified 16 treatment-related symptoms relevant for rectal cancer patients that can be monitored during and after treatment.

## Introduction

Rectal cancer survival rates have improved considerably as a result of earlier detection and improved treatment options. In the Netherlands, 5 year relative survival rates increased from 50% for patients diagnosed in 1989 to 71% for those diagnosed in 2017 [1]. However, rectal cancer treatment contributes to a variety of symptomatic toxicities, including constipation, diarrhea, dyspnea, fatigue, nausea, and pain; all significantly impacting a patient’s health-related quality-of-life (HRQOL) [2, 3]. Lower HRQOL has been associated with increased all-cause mortality in rectal cancer patients [4]. Furthermore, treatment-related symptoms also increase the cost of medical treatment, including additional use of analgesics, prolonged hospital stays, and treatment interruptions [5]. Screening for symptomatic toxicities and intervening timely where possible, are therefore becoming increasingly important.

In current clinical practice, health care providers (HCPs) report treatment-related symptomatic toxicities according to the Common Terminology Criteria for Adverse Events (CTCAE) [6]. Previous studies showed that HCPs often underscore and/or underreport symptom intensity [7–9], resulting in an underestimation of the number and impact of toxicities. Assessing symptomatic toxicities using patient-reported outcomes (PROs) may enhance the precision and comprehensiveness of treatment-related symptom monitoring [10] and may prevent negative downstream consequences by enabling HCPs to respond earlier to symptoms, thereby improving patient-HCP communication and satisfaction [10–13].

Treatment-related symptomatic toxicities can be captured using the PRO-CTCAE, developed by the National Cancer Institute [10]. The PRO-CTCAE includes 78 symptoms that can be reported by patients undergoing systemic therapy, radiotherapy and/or surgery. Implementing the complete PRO-CTCAE in daily clinical practice is considered impractical and burdensome [14], and not all items are relevant for rectal cancer patients. A limited number of items is crucial to measure symptomatic toxicities without bias [15], and may simultaneously achieve greater compliance [16]. Therefore, the objective of this study was to identify a PRO-CTCAE subset for standardized monitoring of treatment-related symptomatic toxicities in rectal cancer patients.

## Methods

**Fig. 1:**
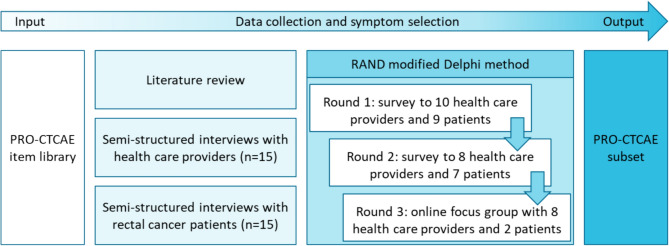
Overview of the study steps for the PRO-CTCAE subset selection. n, number; PRO-CTCAE, Patient-Reported Outcomes version of the Common Terminology Criteria for Adverse Events

The following recommended steps from the ‘Phase 1 guideline for developing Questionnaire Modules’ of the European Organization for Research and Treatment of Cancer (EORTC) Quality of Life group [17] and the RAND modified Delphi procedure [18] were followed to select a PRO-CTCAE subset: (1) a literature review, (2) interviews with HCPs involved in rectal cancer care, (3) interviews with rectal cancer patients, and (4) a Delphi procedure with HCPs involved in rectal cancer care and rectal cancer patient representatives (Fig. 1). The linguistically validated Dutch version of the complete PRO-CTCAE was used as input [19]. The Institutional Review Board of the Netherlands Cancer Institute declared this study outside of the scope of the Dutch Medical Research Involving Human Subjects Act (IRBd19335) and all participants provided informed consent. Local approval was obtained from each participating center.

### Literature review

To identify relevant treatment-related symptomatic toxicities for rectal cancer patients, a PubMed literature review was conducted using a combination of synonyms for ‘rectal cancer’, ‘symptoms’ and ‘treatment’ in the search string (Online Resource 1.1). Observational studies, clinical trials (all phases), and systematic reviews published in Dutch or English were considered eligible for inclusion. To only include publications that reported on symptomatic toxicities of recent treatment modalities, the search was restricted to articles published from 2015 to 2020. Full texts were reviewed if the title and/or abstract reported the prevalence of specific symptoms, and symptoms with ≥ 5% prevalence were extracted and compared with the complete PRO-CTCAE to create a list with all potentially relevant toxicities. The literature search was updated with articles published up to May 2023 to confirm that the identified toxicities were still up to date regarding changes in rectal cancer treatment.

### Semi-structured interviews with HCPs

To ensure input from a heterogeneous, representative population of HCPs from diverse disciplines and medical centers, HCPs involved in rectal cancer care in academic hospitals, community hospitals, and specialized cancer centers in the Netherlands were invited to participate in semi-structured interviews. During these interviews, HCPs completed the full PRO-CTCAE questionnaire, commented on each symptom, and scored the relevance of each symptom on a scale from 1 (not relevant) to 4 (very relevant). HCPs were asked to identify any missing symptoms, and, if so, why the HCP considered this symptom relevant. Lastly, HCPs selected ten symptoms that, based on their professional experience, had the greatest impact on rectal cancer patients and should thus be included in the final subset.

### Semi-structured interviews with patients

A heterogeneous group of rectal cancer patients from one academic hospital, two community hospitals, and one specialized cancer center was invited by their HCP to participate in semi-structured interviews. Purposive sampling was used to enable the selection of a broad range of patients regarding sex, age, treatment type, and disease state [20]. Eligible patients included those treated with curative or palliative intent, with radiotherapy, surgery, chemotherapy, or a combination of these treatments, aged ≥ 18 years, able to read and understand the questionnaire in Dutch or English, and either currently undergoing treatment or having completed treatment within the last 12 months.

At the start of the interview, demographic information was collected using a brief survey, and patients were asked to describe their experiences and relevant symptoms. The relevance and prevalence of these symptoms were further assessed according to the EORTC interview guidelines [17]. Subsequently, patients completed the full PRO-CTCAE questionnaire and scored each symptom’s relevance from 1 (not relevant) to 4 (very relevant). Patients were asked to provide information on experienced symptoms during and after treatment, to identify missing symptoms, to select ten symptoms that had most impacted their HRQOL, and to list the symptoms that should be included or excluded from the subset. Interviews were systematically documented and recorded on audiotape. At the end of the interview, patients were asked for their consent to be contacted for future research questionnaires.

### Literature and interview analyses

An overview table containing symptoms with ≥ 5% prevalence extracted from literature was created, and the proportion of publications that reported these symptoms was calculated. Data from questionnaires completed by HCPs and patients during the semi-structured interviews were analyzed using SPSS (version 27.0, IBM Corporation). Mean relevance scores, mean response scores, frequencies of top ten symptoms, and percentages of HCPs or patients who rated a symptom > 2.00 in relevance were calculated [17].

### RAND modified Delphi procedure

The modified Delphi procedure comprised three rounds: the first two rounds consisted of web-based questionnaires including only PRO-CTCAE symptoms; the third round comprised an online focus group during which PRO-CTCAE symptoms and non-PRO-CTCAE symptoms from the literature and interviews were discussed, and the final symptom subset was selected. After the focus group, this subset was sent to all Delphi participants for final consensus.

### Delphi round 1

HCPs and patients who participated in the interviews and consented to additional questionnaires were invited to participate in the Delphi procedure. Questionnaires were sent and completed through the online survey program Castor [21]. The first Delphi round included only PRO-CTCAE symptoms that were scored ‘relevant’ by at least one HCP or patient during the interviews. Symptoms were accompanied by their mean relevance scores. Participants were asked to classify each symptom as ‘relevant’ or ‘not relevant’. Consensus was set at 75% agreement between HCPs and patients. Results were analyzed using SPSS (version 27.0, IBM Corporation) and aggregated results were used in the second Delphi round.

### Delphi round 2

HCPs and patients from the first Delphi round were invited to participate in the second round. PRO-CTCAE symptoms were accompanied by the percentage of HCPs or patients who scored the symptom as ‘relevant’ in the first round. Symptoms scored as ‘relevant’ or ‘not relevant’ by ≥ 75% of the HCPs and patients in the first round were excluded from the second round. Symptoms scored as ‘relevant’ by ≥ 75% of the HCPs but not by patients were only included in the second round for patients and vice versa. Symptoms scored ‘relevant’ by 26–74% (no consensus) of the patients and HCPs in the first round were included in the second round for both HCPs and patients. In this round, participants again classified symptoms as ‘relevant’ or ‘not relevant’, and aggregated results were used in the third Delphi round.

### Delphi round 3

For the third Delphi round, HCPs and patient representatives were invited for a joint online focus group, moderated by the investigators. This expert panel strived to select one symptom subset specific for rectal cancer patients. PRO-CTCAE symptoms were categorized according to the PRO-CTCAE library guide (gastrointestinal, reproductive and sexual, sleep/wake, mood, urinary, and pain and miscellaneous) [22], and were discussed per category. All 78 PRO-CTCAE symptoms were reviewed, with the total percentages of HCPs or patients who scored a symptom ‘relevant’ in the first two rounds presented for each symptom. Symptoms not included in the PRO-CTCAE (non-PRO-CTCAE symptoms) identified from literature or mentioned by both HCPs and patients during the interviews, were also discussed and added as recommended additional symptoms to monitor if considered relevant by the expert panel.

### Finalization of the symptom subset

The final PRO-CTCAE symptom subset and recommended non-PRO-CTCAE symptoms were presented via Castor to all HCPs and patients from the first Delphi round for formal agreement.

## Results

### Literature review

Following title and abstract screening, and full text review, 21 publications were considered eligible for inclusion; an additional seven publications were included following the literature update (Online Resource 1.2). Review of the publications identified 26 PRO-CTCAE symptoms relevant for rectal cancer patients with a prevalence ≥ 5% (Online Resource 1.3). The following treatments were investigated in included publications (excluding systematic reviews): radiotherapy (26% of publications), chemotherapy (65% of publications), chemoradiation (48% of publications), surgery (91% of publications) and immunotherapy (13% of publications). The most frequently reported PRO-CTCAE symptoms were ‘diarrhea’ (64% of publications), ‘nausea’ (39% of publications), ‘vomiting’ (36% of publications), ‘fatigue’ (29% of publications) and ‘rash’ (25% of publications). The following non-PRO-CTCAE symptoms were also frequently reported with ≥ 5% prevalence: ‘proctitis’ (25% of publications), ‘cystitis’ (18% of publications), and ‘stomatitis’ (14% of publications). In the literature update, ‘fever’, ‘flu-like symptoms’, ‘weight loss’, ‘clustering of stools’, ‘defecation frequency or bowel movement frequency’, and ‘syncope’ were additional identified symptoms with ≥ 5% prevalence in one or more publications.

### Semi-structured interviews with HCPs

Fifteen HCPs from community hospitals (n = 4 HCPs), academic hospitals (n = 3 HCPs) and specialized cancer centers (n = 8 HCPs) in the Netherlands participated in the semi-structured interviews. The sample consisted of seven (47%) radiation oncologists, four (27%) surgeons, three (20%) medical oncologists, and one (7%) nurse specialist. HCPs had varying work experience ranging from 3 years till 27 years. HCPs considered the symptoms ‘fecal incontinence’ (mean score 3.93), ‘diarrhea’ (mean score 3.80), ‘fatigue’ (mean score 3.67) and ‘general pain’ (mean score 3.53) most relevant (Online Resource 1.4). Other symptoms with a mean relevance score > 3.00 were ‘ability to achieve and maintain erection’, ‘constipation’, ‘pain in the abdomen’, ‘pain during vaginal sex’, ‘decreased appetite’, ‘ejaculation problems’, ‘urinary incontinence’, ‘decreased libido’, ‘nausea’, ‘concentration’, ‘insomnia’, ‘anxiety’ and ‘frequent urination’. When analyzing mean relevance scores according to treatment provided by HCPs, ‘fecal incontinence’ and ‘general pain’ scored ≥ 3.50 among all treatment modalities (Online Resource 1.5). With the exception of ‘mouth/throat sores’, all HCP top ten symptoms had a mean relevance score ≥ 2.00. Symptoms with a mean relevance score of 4.0 were ‘fecal incontinence’ among HCPs working with radiotherapy or surgery, and ‘fatigue’ and ‘general pain’ among HCPs working with chemotherapy. ‘Diarrhea’, ‘concentration’ and ‘hand-foot syndrome’ were a top ten symptom for all HCPs involved in chemotherapy. ‘Diarrhea’ and ‘ability to achieve and maintain erection’ were top ten symptoms for all HCPs with experience in surgery.

### Semi-structured interviews with rectal cancer patients

Fifteen rectal cancer patients participated in the semi-structured interviews. The median age was 61 years (range 52–78). Most patients were male (73%), had completed higher education (53%), and had completed primary treatment (67%, Table 1).

**Table 1:** Characteristics of rectal cancer patients participating in the semi-structured interviews

	n (%)
Age in years, median (range)	61 (52–78)
Sex	
Male	11 (73)
Female	4 (27)
Completed level of education	
Low	5 (33)
Middle	2 (13)
High	8 (53)
Disease stage	
I	1 (7)
II	5 (33)
III	4 (27)
IV	5 (33)
Type of care	
Curative	8 (53)
Palliative	7 (47)
Stoma	
No	9 (60)
Yes, temporary	3 (20)
Yes, permanent	3 (20)
Treatment characteristics	
Chemotherapy only	1 (7)
Radiotherapy only	2 (13)
Radiotherapy and surgery	2 (13)
Chemoradiation	4 (27)
Chemoradiation and surgery	6 (40)
Treatment status at time of interview	
On treatment	5 (33)
< 1 month post treatment	2 (13)
1–3 months post treatment	5 (33)
3–6 months post treatment	2 (13)
> 6 months post treatment	1 (7)

Percentages may not total 100 because of rounding

*n* number

From the patient’s perspective, ‘fatigue’ was considered the most relevant symptom with a mean score of 2.27. ‘Bloating of the abdomen’ and ‘pain during vaginal sex’ both had mean relevance scores of 2.00, while all other symptoms had mean scores < 2.00 (Online Resource 1.4). ‘Fatigue’ was most frequently mentioned (7 times) as a top ten symptoms impacting patients’ HRQOL, followed by ‘pain in the abdomen’ and ‘frequent urination’ (both mentioned 4 times). When analyzing the top ten symptoms per treatment modality, ‘fatigue’ was mentioned by at least three patients in each treatment modality (Table 2). ‘Pain in the abdomen’, ‘decreased appetite’, ‘decreased libido’, ‘inability to reach orgasm’ and ‘numbness and tingling’ were mentioned by at least two patients per treatment modality. The only symptom with a mean relevance score ≥ 2.00 across all treatment modalities was ‘pain during vaginal sex’, with a score of 2.50 for patients treated with radiotherapy and/or surgery and a score of 2.00 among patients treated with chemotherapy.

**Table 2:** Mean relevance score and number of times a PRO-CTCAE symptom was mentioned as part of the top ten during the semi-structured interviews with patients, according to treatment

PRO-CTCAE symptom	Treatment modality^a^					
	Radiotherapy (n = 14)		Surgery (n = 8)		Chemotherapy (n = 11)	
	Mean relevance score	times in top 10	Mean relevance score	times in top 10	Mean relevance score	times in top 10
Gastrointestinal disorders						
Pain in the abdomen^b^	1.50	4	1.25	2	1.36	3
Bloating of the abdomen^b^	**2.07**	1	1.63	0	**2.00**	1
Constipation^b^	1.50	2	1.25	1	1.36	1
Diarrhea^b^	1.64	2	1.75	1	1.55	2
Taste changes	1.57	1	1.25	1	1.55	1
Decreased appetite	1.57	2	1.63	2	1.45	3
Fecal incontinence^b^	1.36	1	1.25	1	1.55	2
Flatulence	1.50	2	1.38	1	1.45	2
Nausea	1.36	0	1.25	0	1.55	1
Reproductive and sexual disorders						
Pain during vaginal sex^b,c^	**2.50**	1	**2.50**	1	**2.00**	1
Ability to achieve and maintain erection^b^	1.86	2	1.50	1	1.55	1
Decreased libido^b^	1.71	3	1.88	2	1.64	2
Inability to reach orgasm	1.93	3	**2.00**	2	1.73	2
Delayed orgasm	**2.00**	1	**2.00**	1	1.73	1
Renal and urinary disorders						
Frequent urination	1.57	3	1.38	1	1.55	3
Urinary incontinence^b^	1.00	0	1.00	0	1.18	1
Painful urination^b^	1.36	2	1.00	0	1.00	0
Urinary urgency	1.43	0	1.25	0	1.45	1
Sleep/wake and mood						
Fatigue^b^	**2.29**	6	1.88	3	**2.00**	5
Insomnia	1.86	3	1.13	0	1.62	1
Anxiety^b^	1.43	1	1.00	0	1.27	0
Neurological disorders, memory, and concentration						
Numbness and tingling	1.64	2	1.75	2	1.55	3
Memory	1.21	2	1.25	1	1.27	2
Concentration	1.50	2	1.50	1	1.50	2
Pain, mouth, dermatological, and respiratory disorders						
General pain^b^	1.43	1	1.38	1	1.55	1
Mouth/throat sores	1.36	1	1.25	1	1.45	1
Difficulty swallowing	1.29	1	1.50	1	1.27	2
Hand-foot syndrome^b^	1.29	1	1.13	1	1.36	1
Itching skin	1.36	1	1.50	1	1.36	1
Shortness of breath	1.14	1	1.00	0	1.00	0

Only symptoms that were mentioned at least once as top ten symptoms that most impacted a patients HRQOL are shown. Bold indicates a mean relevance score ≥ 2.00 Patients completed the PRO-CTCAE questionnaire and scored each item for relevance on a scale from 1 (not relevant) to 4 (very relevant) and were asked to choose a maximum of ten symptoms that had most impacted their HRQOL

^a^Treatment combinations possible; one patient received chemotherapy only, two patients received radiotherapy only, two patients received radiotherapy and surgery, four patients received chemoradiation and six patients received chemoradiation with surgery

^b^Symptom included in final PRO-CTCAE subset

^c^Symptom only scored by n = 3 female patients

*PRO-CTCAE* Patient reported outcomes version of the common terminology criteria for adverse events, *HRQOL* Health-related quality of life

### Delphi procedure

Ten HCPs (67%) and nine patients (90%) participated in the first Delphi round. Consensus for inclusion in the PRO-CTCAE subset was achieved for ‘diarrhea’ and ‘fatigue’ (Table 3). Forty-two symptoms were scored ‘not relevant’ by ≥ 75% of the participants and placed on the exclusion list for further review in the third round. Thirteen symptoms were scored ‘relevant’ or ‘not relevant’ by ≥ 75% of the HCPs but not by patients and were only included in the next questionnaire round for patients. Nine symptoms were scored ‘not relevant’ by ≥ 75% of patients but not HCPs and were only included in the next questionnaire round for HCPs. Eight symptoms were scored ‘relevant’ by 26–74% of HCPs and patients and were transferred to the next questionnaire round for both HCPs and patients.

**Table 3:**
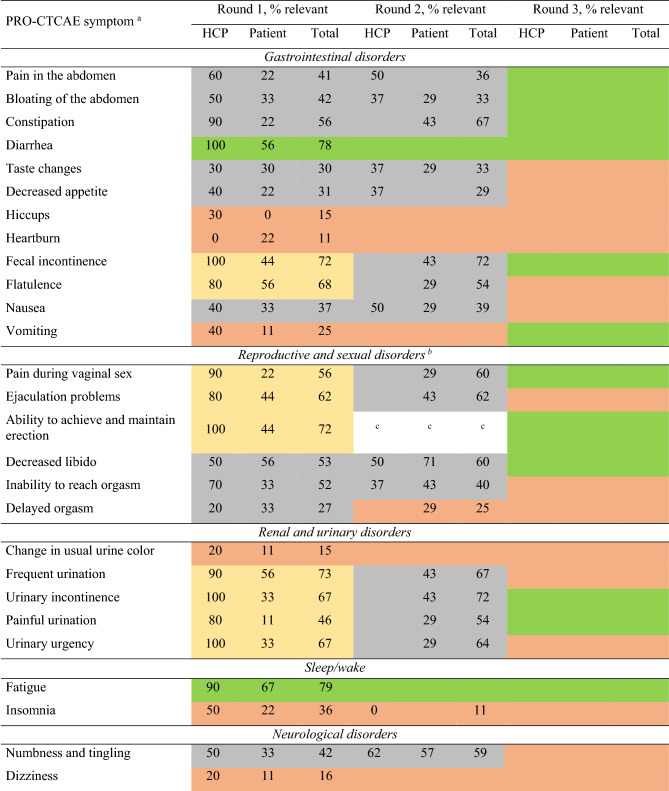

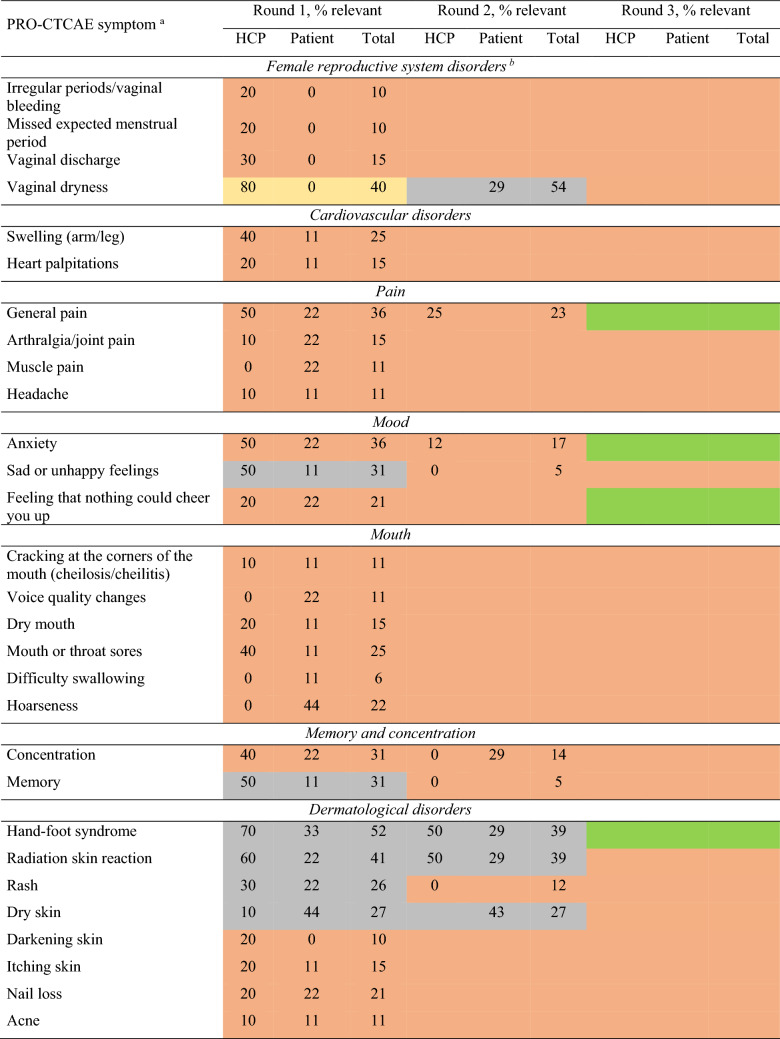

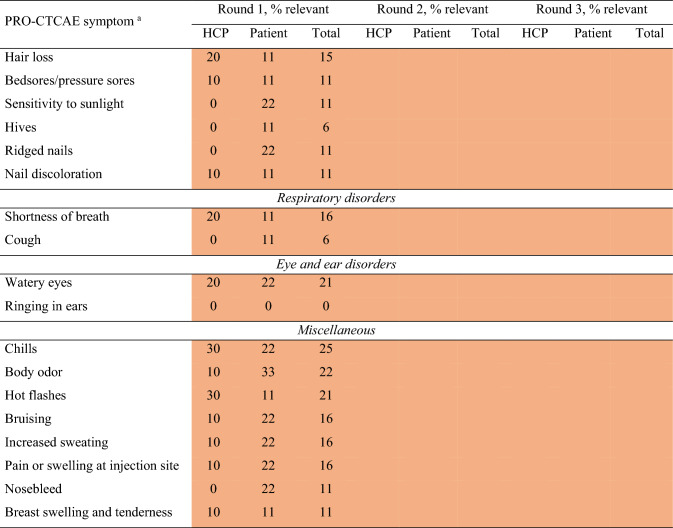
Percentage agreement to include symptoms in round 1–3 of the Delphi procedure for health care providers and patients

Ten HCPs and nine patients participated in round 1, eight HCPs and seven patients participated in round 2, and eight HCPs and two patient representatives participated in round 3. Numbers in each cell present the percentage of participants who scored a symptom ‘relevant’. If in round 1, a symptom was scored ‘relevant’ by ≥ 75% of HCPs, but not patients, the symptom was only asked in the second round of the patient survey, and vice-versa. If in round 1 a symptom was scored ‘relevant’ by 26–74% of HCPs and patients, the symptom was asked in both HCP and patient questionnaires in the second round. Cells in round three are empty as no formal scoring took place (online focus group meeting)

Green: total agreement score of both HCPs and patients was ≥ 75%, item was included; yellow: either ≥ 75% of HCPs or patients scored item as relevant, total agreement score not ≥ 75%, item was not (yet) included; orange: both ≤ 25% of HCPs and patients scored item as relevant, item was excluded; grey: both 25–75% patients and HCP scored item as relevant, item was not (yet) included

^a^The symptoms ‘wheezing’, stretch marks’, ‘blurred vision’, ‘light flashes’, ‘visual floaters’ and ‘decreased sweating’ are not displayed in the table. These symptoms were not included in Delphi round 1 and have no scores available

^b^Both male and female patients scored sex-specific symptoms. Numbers in each cell present the percentage of participants who scored a symptom ‘relevant’, if a patient reported that a symptom was ‘not applicable’, this patient was still considered for the percentage calculation

^c^Symptom was not included properly, no scores available for this round

*HCP* Health care provider, *PRO-CTCAE* Patient reported outcomes version of the common terminology criteria for adverse events

Eight HCPs (80%) and seven (78%) patients participated in the second Delphi round. Consensus for inclusion was not reached for any symptom, and therefore no symptoms were added in this round. Eight symptoms were considered ‘not relevant’ by ≥ 75% of the participants and excluded, resulting in a total of 50 excluded symptoms. Consensus was not reached for 21 symptoms, which were transferred to the third round (Table 3).

Eight HCPs (three radiation oncologists, three surgeons, one medical oncologist and one nurse specialist) and two patient representatives participated in Delphi round three. The participants unanimously agreed to include the two symptoms that had been added after the first two Delphi rounds. HCPs argued that only symptoms for which they could provide solutions (e.g., medication or referral) should be included in the final subset. From the symptoms for which no consensus was reached, ‘fecal incontinence’, ‘constipation’ and ‘urinary incontinence’ were included due to their high scores from HCPs in the first two Delphi rounds. HCPs explained that they scored ‘flatulence’ as relevant because they believed it to be unpleasant for patients, but after discussion, ‘bloating of the abdomen’ was deemed clinically more relevant and therefore preferred over ‘flatulence’. ‘Vomiting’, ‘pain in the abdomen’, ‘painful urination’ and ‘hand-foot syndrome’ were included as these symptoms were indicative of medical problems that may require (adaptations in) treatment.

Patient representatives emphasized including symptoms of the reproductive and sexual disorder category. ‘Decreased libido’ was included given its relevance to both males and females. ‘Ejaculation problems’ was exchanged with ‘ability to achieve and maintain erection’ as the latter was scored more relevant by HCPs and patients in the interviews. ‘Pain during vaginal sex’ was included to cover a female-specific sexual disorder symptom. Even though the symptoms ‘anxiety’, ‘feeling that nothing could cheer you up’ and ‘general pain’ were not scored relevant by most HCPs nor by patients during the first two Delphi rounds, the focus group decided to include these symptoms to cover patients’ emotional wellbeing and provide an opportunity to ask further questions. Patient representatives argued that including rare symptoms might cause unnecessary anxiety for rectal cancer patients, and the focus group agreed on excluding remaining symptoms scored ‘relevant’ by a small percentage (≤ 25%) of HCPs and patients.

Treatment-specific symptoms were also discussed, with ‘numbness and tingling’ and ‘rash’ being recommended for inclusion in the subset for chemotherapy patients. Of the non-PRO-CTCAE symptoms from literature or mentioned during interviews, ‘weight loss’, ‘rectal bleeding’, ‘defecation urge’ and ‘chest pain’ were recommended to include due to their potential indication of medical problems and association with critical conditions (e.g., chest pain as indication of coronary artery vasospasms induced by 5-fluorouracil and/or capecitabine). ‘Pain during defecation’ was recommended because patient representatives stressed that not all patients may consider this specific symptom when only a ‘general pain’ question is included.

The final PRO-CTCAE subset is presented in Table 4 and Online Resource 2. The final set with PRO-CTCAE symptoms and recommended non-PRO-CTCAE symptoms was presented to all Delphi participants for formal agreement (overall response 79%; 8/10 HCPs and 7/9 patients). Seven HCPs (87%) and seven patients (100%) agreed with the final PRO-CTCAE subset. Six HCPs (75%) and seven patients (100%) agreed with the recommended non-PRO-CTCAE symptoms: one HCP disagreed with the inclusion of ‘chest pain’ and ‘weight loss’ and considered both terms not specific enough; and one HCP disagreed with the inclusion of ‘chest pain’ without providing a specific reason.

**Table 4:** Final PRO-CTCAE symptom subset and additionally recommended non-PRO-CTCAE items

PRO-CTCAE subset	Recommended non-PRO-CTCAE symptoms	Recommended PRO-CTCAE symptoms per treatment modality
Gastrointestinal disorders		
• Diarrhea	• Rectal bleeding	
• Fecal incontinence	• Pain during defecation	
• Constipation	• Defecation urge	
• Bloating of the abdomen		
• Pain in the abdomen		
• Vomiting		
Reproductive and sexual disorders		
• Decreased libido		
• Pain during vaginal sex		
• Ability to achieve and maintain erection		
Sleep/wake		
• Fatigue		
Mood		
• Anxiety		
• Feeling that nothing could cheer you up		
Renal and urinary disorders		
• Urinary incontinence		
• Painful urination		
Pain, dermatological disorders, and miscellaneous		
• General pain	• Weight loss	• Numbness and tingling (chemotherapy) • Rash (chemotherapy)
• Hand-foot syndrome	• Chest pain	

*PRO-CTCAE* Patient reported outcomes version of the common terminology criteria for adverse events

## Discussion

Based on a comprehensive mixed methods approach including a literature review, and qualitative input from both HCPs and patients, we selected a PRO-CTCAE subset for monitoring symptoms in rectal cancer patients during treatment and follow-up. The PRO-CTCAE subset consists of 16 symptoms: ‘diarrhea’, ‘fecal incontinence’, ‘constipation’, ‘bloating of the abdomen’, ‘pain in the abdomen’, ‘vomiting’, ‘decreased libido’, ‘pain during vaginal sex’, ‘ability to achieve and maintain erection’, ‘fatigue’, ‘anxiety’, ‘feeling that nothing could cheer you up’, ‘urinary incontinence’, ‘painful urination’, ‘general pain’, and ‘hand-foot syndrome’. Five non-PRO-CTCAE symptoms were added to the recommended section: ‘rectal bleeding’, ‘pain during defecation’, ‘defecation urge’, ‘weight loss’, and ‘chest pain’. Using this PRO-CTCAE subset will provide a more comprehensive overview of patient-reported symptoms, thereby contributing to optimizing (follow-up) care for rectal cancer patients.

Over the last years, the importance of PROs for symptom management in clinical practice has become increasingly recognized. Standardized PRO monitoring in clinical practice contributes to optimizing the response to symptoms experienced by cancer patients and thereby improves long-term HRQOL and overall survival [11, 23]. Moreover, monitoring PROs enables HCPs to respond earlier to evolving symptoms with, for example, dose modifications or timely initiation of supportive measures, which may therefore result in a more effective response [24, 25]. For treatment options with similar disease-free survival [26], PRO data can inform decision making by HCPs and patients [2]. To enable standardized symptom monitoring, PRO-CTCAE subsets have previously been identified for lung cancer [19] and prostate cancer [27], but had not yet been established for rectal cancer.

The selected PRO-CTCAE subset for rectal cancer was approved by 87% of HCPs and 100% of patients. During the focus group, patient representatives argued that symptoms unlikely to occur in most rectal cancer patients should not be included in the subset, as their presentation may cause unnecessary anxiety. HCPs emphasized including symptoms indicative of medical problems and symptoms for which solutions could be provided. Previous research among HCPs and patients in routine dialysis care has shown a similar response from HCPs; while patients in this study indicated that explanations about symptoms are desired, even when there are no treatment options available [28]. This difference in scoring symptom significance between HCPs and patients, and between patients with different diseases, emphasizes the importance of including the perspectives of both HCPs and patients, and patients with relevant disease experience, when selecting a symptom subset for clinical monitoring.

We aimed to identify one core-PRO-CTCAE subset for standardized symptom monitoring in rectal cancer, which can be used irrespective of treatment type and phase. The final subset includes 16 PRO-CTCAE symptoms. While this selected number of symptoms is less than the full PRO-CTCAE, frequent monitoring of these symptoms might still be burdensome, and not all symptoms may be relevant for each specific treatment. However, by monitoring one consistent PRO-CTCAE subset for all patients during treatment and follow-up, data collection and analysis will become more efficient and less variable; time and effort needed for training staff, developing and updating data collection tools, and analyzing the results will reduce over time. Furthermore, using the same PRO-CTCAE subset during treatment and follow-up provides patients with a consistent and reliable means of reporting their symptoms, which can result in better patient engagement, as patients can track their symptoms over time and understand the impact of their treatments more clearly. The feasibility of PRO-CTCAE symptom monitoring among rectal cancer patients via an electronic survey was evaluated in the PROSPECT trial [29, 30]. In this trial the authors found that, during active treatment, most participants were willing and able to report information on 15 PRO-CTCAE symptoms every week. Selecting a symptom subset for standardized symptom monitoring in rectal cancer patients was not part of the objectives of the PROSPECT trial [31]. The symptoms monitored in this trial were selected based on expected symptomatic toxicities related to trial therapies, and on previously identified prevalent symptoms among patients with diverse cancer types who were undergoing treatment. Therefore, not all included symptoms may have been relevant for rectal cancer patients specifically. Furthermore, symptoms related to urinary or reproductive and sexual disorders were not included. Six PRO-CTCAE symptoms (‘anxiety’, ‘constipation’, ‘fatigue’, ‘diarrhea’, ‘general pain’ and ‘vomiting’) that were evaluated in the PROSPECT trial were also included in our final PRO-CTCAE subset. As the PROSPECT trial is still ongoing, the effect of weekly monitoring of the PRO-CTCAE symptoms has not yet been evaluated.

Strengths of our study comprise the inclusion of a heterogeneous patient population treated with various treatment modalities with both curative and palliative intent. This study population, therefore, allowed us to establish one symptom-subset applicable to most rectal cancer patients, irrespective of treatment and follow-up phase. ‘Numbness and tingling’ and ‘rash’ were included specifically for patients receiving chemotherapy to address treatment-specific symptoms [32], while keeping the core-subset relatively small and therefore easier to use in daily clinical practice. Furthermore, the symptom subset was established with input from HCPs from diverse disciplines and medical centers, which may facilitate the implementation of the subset in clinical care [24], as it is more likely that HCPs from the full spectrum of rectal cancer care perceive the subset as valuable.

Our study has some limitations. During the literature study update, six additional non-PRO-CTCAE symptoms with ≥ 5% prevalence were identified. Of these symptoms, ‘defecation frequency’ and ‘weight loss’ were both mentioned by HCPs and/or patients during the interviews and discussed in the focus group. The other ‘newly’ identified symptoms (‘fever’, ‘flu-like symptoms’, ‘clustering of stools’, and ‘syncope’) were only reported in one study and not mentioned during the interviews and were therefore not discussed in the focus group. However, the standardized PRO-CTCAE forms include the option to report ‘other symptoms’ [22], which enables patients to report symptoms that are not included in the subset. Secondly, 73% of the included patients was male and 53% had completed higher education. Lower educational levels have been associated with worse health literacy [33] and high symptom burden in one-year cancer survivors [34]. The inclusion of few female patients and few patients with lower educational levels may have affected which symptoms were included in the subset. To evaluate if results are also applicable and perceived valuable by broader populations of rectal cancer patients and HCPs, psychometric validation, and assessment of the effectiveness and user compliance over time of this PRO-CTCAE subset is recommended.

To our knowledge, this study represents the first effort to identify a PRO-CTCAE-based subset for symptom monitoring in rectal cancer patients. Previous literature and the perspectives of both HCPs and patients were considered. A core-subset of 16 symptomatic toxicities was selected, treatment-specific symptomatic toxicities can be included easily using the PRO-CTCAE form builder [22]. Patients from different treatment phases were represented in the study, making the set applicable to a broad rectal cancer patient population. Psychometric validation and assessment of the effectiveness of PRO symptom monitoring in rectal cancer patients in an (inter)national setting, and determination of the optimal frequency for use in clinical practice are recommended. Standardized symptom monitoring using this PRO-CTCAE subset is a starting point for improving quality of care and incorporating rectal cancer patients’ perspectives in a multidisciplinary setting.

## Supplementary Information

Below is the link to the electronic supplementary material.

Supplementary file1 (DOCX 63 KB)

Supplementary file2 (DOCX 28 KB)

## Data Availability

The datasets generated and analyzed during the current study are not publicly available because individual privacy could be compromised, but aggregated de-identified data are available from the corresponding author by reasonable request.
