# Supplementary material for: Using a modified Delphi procedure to select a PRO-CTCAE-based subset for patient-reported symptomatic toxicity monitoring in rectal cancer patients
Source: Qual Life Res. 2024 Sep 8;33(11):3013–26. doi: 10.1007/s11136-024-03767-0 (PMC11541275; doi:10.1007/s11136-024-03767-0)
Supplement: Supplementary file 1 — Supplementary file1 (DOCX 63 KB) [file 11136_2024_3767_MOESM1_ESM.docx]

**Online Resources 1**

Article title: *Using a modified Delphi procedure to select a PRO-CTCAE-based subset for patient-reported symptomatic toxicity monitoring in rectal cancer patients.*

Journal: *Quality of Life Research*

Authors: *Yvonne M. Geurts, Femke Peters, Esther Feldman, Jeanine Roodhart, Milan Richir, Jan Willem T. Dekker, Geerard Beets, Jeltsje S. Cnossen, Patricia Bottenberg, Martijn Intven, Marcel Verheij, Kelly M. de Ligt, Iris Walraven.*

Corresponding author: *Yvonne M. Geurts, Department of IQ Health, Radboud university medical center, The Netherlands,* [*yvonne.m.geurts@radboudumc.nl*](mailto:yvonne.m.geurts@radboudumc.nl)

|  | Page |
| --- | --- |
| *Online Resource 1.1*  Search string for literature review. | 2 |
| *Online Resource 1.2*  Reference information of eligible studies from the literature review. | 3 |
| *Online Resource 1.3*  Symptoms among rectal cancer patients according to literature. | 7 |
| *Online Resource 1.4*  Mean relevance scores for each PRO-CTCAE symptom scored by HCPs and patients during the interview rounds. | 10 |
| *Online Resource 1.5*  Number of times a PRO-CTCAE symptom was mentioned as part of the top ten during the semi-structured interviews with HCPs, according to treatment. | 13 |

| **Online Resource 1.1. Search string for literature review** | |
| --- | --- |
| Main topic | PubMed search terms |
| Rectal cancer | ("Rectal Neoplasms"[MeSH Terms] OR (("rectum"[Title] OR "rectal"[Title]) AND ("cancer"[Title/Abstract] OR "carcino*"[Title/Abstract] OR "tumo*"[Title/Abstract] OR "neoplasm*"[Title/Abstract])))  AND |
| Symptoms | ("General Surgery"[MeSH Terms] OR "Drug Therapy"[MeSH Terms] OR "Therapeutics"[MeSH Terms] OR "radiotherapy"[MeSH Terms] OR "chemotherapy, adjuvant"[MeSH Terms] OR "Antineoplastic Combined Chemotherapy Protocols"[MeSH Terms] OR "therapy"[Title/Abstract] OR "treatment"[Title/Abstract] OR "surger*"[Title/Abstract] OR "operation"[Title/Abstract] OR "surgical"[Title/Abstract] OR "operative"[Title/Abstract] OR "resect*"[Title/Abstract] OR "excision*"[Title/Abstract] OR "radiation"[Title/Abstract] OR "radio*"[Title/Abstract] OR "chemo*"[Title/Abstract])  AND |
| Treatment | ("therapeutics/adverse effects"[MeSH Terms] OR "Drug-Related Side Effects and Adverse Reactions"[MeSH Terms] OR "Long Term Adverse Effects"[MeSH Terms] OR "adverse effect"[Title] OR "adverse event"[Title] OR "toxic*"[Title] OR "complic*"[Title] OR "side effect"[Title] OR "contraindicat*"[Title]) |

| **Online Resource 1.2. Reference information of eligible studies from the literature review.** | | | |
| --- | --- | --- | --- |
| Authors | Year | Title | Journal |
| Ansari, et al. | 2017 | Acute adverse events and postoperative complications in a randomized trial of preoperative short-course radiotherapy versus long-course chemoradiotherapy for T3 adenocarcinoma of the rectum | Ann Surg 265:882-888. |
| Bach, et al. | 2021 | Radical surgery versus organ preservation via short-course radiotherapy followed by transanal endoscopic microsurgery for early-stage rectal cancer (TREC): a randomised, open-label feasibility study | Lancet Gastroenterol Hepatol 6:92-105. |
| Bazarbashi, et al. | 2022 | Neoadjuvant concurrent chemotherapy using infusional gemcitabine in locally advanced rectal cancer: A phase II trial | Cancer Medicine 11:2056-2066. |
| Charkavarthy, et al. | 2020 | Intergroup randomized phase III study of postoperative oxaliplatin, 5-fluorouraccil, and leucovorin versus oxaliplatin, 5-fluorouracil, leucovorin, and bevacizumab for patients with stage II or III rectal cancer receiving preoperative chemoradiation: a trial of the ECOG-ACRIN research group (E5204) | The Oncologist 25:e798-e807. |
| Chen, et al. | 2023 | Neoadjuvant PD-1 blockade with sintilimab in mismatch repair deficient, locally advanced rectal cancer: an open-label, single-centre phase 2 study | Lancet Gastoenterol Hepatol 8:422-31 |
| Ding, et al. | 2022 | mFOLFOXIRI versus mFOLFOX6 as neoadjuvant chemotherapy in locally advanced rectal cancer: A Propensity Score Matching Analysis | Clinical Colorectal Cancer 21:e12-e20. |
| Eisterer, et al. | 2017 | Neoadjuvant chemotherapy with capecitabine, oxaliplatin and bevacizumab followed by concomitant chemoradiation and surgical resection in locally advanced rectal cancer with high risk of recurrence - a phase II study | Anticancer research 37: 2683-2691. |
| Jo, et al. | 2023 | A Phase II Study of Preoperative Chemoradiotherapy with Capecitabine Plus Simvastatin in Patients with Locally Advanced Rectal Cancer | Cancer Res Treat 55(1):189-195 |
| Koike, et al. | 2017 | Efficacy and safety of neoadjuvant chemotherapy with oxaliplatin, 5-fluorouracil, and levofolinate for T3 or T4 stage II/III rectal cancer: the FACT trial | Cancer Chemother Pharmacol 79:519-525. |
| Konishi, et al. | 2019 | Phase II trial of neoadjuvant chemotherapy, chemoradiotherapy, and laparoscopic surgery with selective lateral node dissection for poor-risk low rectal cancer | Ann Surg Oncol 26:2507-2513. |
| Liu, et al. | 2021 | Postoperative complications observed with robotic versus laparoscopic surgery for the treatment of rectal cancer: An updated meta-analysis of recently published studies | Medicine 100:36. |
| Meada, et al. | 2018 | Neoadjuvant radiotherapy with capecitabine plus bevacizumab for locally advanced lower rectal cancer: results of a single-institute phase II study | Anticancer research 38: 4193-3197. |
| Paszt, et al. | 2022 | Clinical benefits of oral capecitabine over intravenous 5-fluoracyl regimen in case of neoadjuvant chemoradiotherapy followed by surgery for locally advanced rectal cancer | Pathol Oncol Res 28:1610722. |
| Pinto, et al. | 2018 | Phase II study of preoperative treatment with external radiotherapy plus panitumumab in low-risk locally advanced rectal cancer (RaP study/STAR-03) | The Oncologist 23:912-918. |
| Shamseddine, et al. | 2020 | Efficacy and safety‑in analysis of short‑course radiation followed by mFOLFOX‑6 plus avelumab for locally advanced rectal adenocarcinoma | Radiat Oncol 15:233. |
| Sipaviciute, et al. | 2020 | Late gastrointestinal toxicity after radiotherapy for rectal cancer: a systematic review | Int J Colorectal Dis 35:977-983. |
| Toritani, et al. | 2020 | A prospective, single-arm, multicenter trial of neoadjuvant chemotherapy with mFOLFOX6 plus panitumumab without radiotherapy for locally advanced rectal cancer. | Int J Colorectal Dis 35:2197-2204. |
| Tsukamoto, et al. | 2017 | Surgical outcomes of robot-assisted rectal cancer surgery using the da Vinci Surgical System: a multi-center pilot phase II study | JJCO 47(12):1135-1140. |
| Ueki, et al. | 2016 | A feasibility study of neoadjuvant XELOX without radiotherapy for locally advanced lower rectal cancer | Anticancer Research 36: 741-748 |
| Velenik, et al. | 2020 | Influence of concurrent capecitabine based chemoradiotherapy with bevacizumab on the survival rate, late toxicity and health-related quality of life in locally advanced rectal cancer: a prospective phase II CRAB trial | Radiol Oncol 54(4): 461-469. |
| Verweij, et al. | 2023 | Patient- and physician-reported radiation-induced toxicity of short-course radiotherapy with a prolonged interval to surgery for rectal cancer | Colorectal Disease 25:24-30. |
| Wang, et al. | 2020 | The severity of postoperative complications after robotic versus laparoscopic surgery for rectal cancer: A systematic review, meta-analysis and meta-regression | PLOS One. |
| Wang, et al. | 2022 | High dose chemoradiotherapy increases chance of organ preservation with satisfactory functional outcome for rectal cancer. | Radiation Oncology 17:98 |
| Wee, et al. | 2018 | Intensity-modulated radiotherapy versus three-dimensional conformal radiotherapy in rectal cancer treated with neoadjuvant concurrent chemoradiation: a meta-analysis and pooled-analysis of acute toxicity | JJCO 48(5):458-466. |
| Yamada, et al. | 2022 | Carbon Ion Radiotherapy for Locally Recurrent Rectal Cancer of Patients with Prior Pelvic Irradiation | Ann Surg Oncol 29:99-106. |
| Yamazaki, et al. | 2022 | Galunisertib plus neoadjuvant chemoradiotherapy in patients with locally advanced rectal cancer: a single-arm, phase 2 trial | Lancet Oncol 23:1189-200. |
| Zhou, et al. | 2021 | The efficacy and safety of adding bevacizumab in neoadjuvant therapy for locally advanced rectal cancer patients: a systematic review and meta-analysis | Transl Oncol 14(1):100964. |
| Zimmerman, et al. | 2022 | Acute toxicities of patients with locally advanced rectal cancer treated with intensified chemoradiotherapy within the CAO/ARO/AIO-12 trial: comparing conventional versus VMAT planning at a single center | Sci Rep 12:21263. |

| **Online Resource 1.3. Symptoms among rectal cancer patients according to literature** | | | | | | |
| --- | --- | --- | --- | --- | --- | --- |
| First author, year | | Study population | | Study treatments | | Symptoms with ≥5% prevalence ^b^ |
| Ansari, 2017 | | 317 | | RT and surgery | | Radiation dermatitis, diarrhea, proctitis ^a^, pain due to radiation, dysuria, urinary frequency, perineal pain ^a^, stomatitis/pharyngitis ^a^, hand-foot syndrome, nausea, vomiting, fatigue |
| Bach, 2021 | | 123 | | RT and surgery or surgery alone | | Cystitis ^a^, diarrhea, nausea, proctitis ^a^, skin reaction |
| Bazarbashi, 2022 | | 40 | | CRT, surgery, and chemotherapy | | Nausea, vomiting, stomatitis ^a^, diarrhea, pain in the abdominal pain, anorexia, proctitis ^a^, constipation, fatigue, cystitis ^a^, skin desquamation ^a^ |
| Charkavarthy, 2020 | | 347 | | CRT, surgery, and chemotherapy | | Fatigue, anorexia, dehydration ^a^, diarrhea, nausea, vomiting, neuropathy, pain musculoskeletal |
| Chen, 2023 | | 17 | | Immunotherapy and surgery with/without chemotherapy | | Fatigue, fever ^a^, cough, flu-like symptoms ^a^, diarrhea, vomiting, constipation, intestinal obstruction ^a^, skin rash, neuropathy |
| Ding, 2022 | | 312 | | Chemotherapy and surgery | | Nausea/vomiting |
| Eisterer, 2017 | | 25 | | Chemotherapy, CRT, and surgery | | Diarrhea, mucositis, skin reaction, ileus ^a^, nausea, vomiting |
| Jo, 2023 | | 53 | | CRT, surgery, and chemotherapy | | Nausea, anorexia, diarrhea, abdominal pain, hand-foot syndrome |
| Konishi, 2019 | | 43 | | Chemotherapy, CRT, and surgery | | Diarrhea |
| Meada, 2018 | | 25 | | CRT and surgery | | Diarrhea, hand-foot syndrome, anal pain ^a^ |
| Paszt, 2022 | | 185 | | CRT and surgery | | Diarrhea, cystitis ^a^, proctitis ^a^, weight loss ^a^, nausea/vomiting, intestinal passage disorder ^a^ |
| Pinto, 2018 | | 98 | | Immunotherapy, RT, surgery, and chemotherapy | | Diarrhea, stomatitis ^a^, abdominal pain, skin rash, nail problems, asthenia ^a^, radiodermatitis |
| Shamseddine, 2020 | | 13 | | RT, chemotherapy, and surgery | | Diarrhea, nausea, abdominal distention, vomiting, chills, fatigue, insomnia, upper respiratory tract infection ^a^, dry skin, rash |
| Sipaviciute, 2020 | | 2115 | | RT combined with other treatments | | Diarrhea, fecal incontinence, incontinence to gas, rectal bleeding ^a^, rectal pain ^a^, obstruction ^a^, mucus in stools ^a^, blood in stools ^a^, anal stricture ^a^ |
| Toritani, 2020 | | 50 | | Chemotherapy, immunotherapy, and surgery | | Stomatitis ^a^, anorexia, skin toxicity, anastomotic leakage ^a^, paralytic ileus ^a^, transient urinary retention ^a^ |
| Ueki, 2016 | | 29 | | Chemotherapy and surgery with/without CT | | Nausea, vomiting, diarrhea, anorexia, mucositis, neuropathy, hand-foot syndrome, fatigue |
| Velenik, 2020 | | 60 | | CRT, surgery, and chemotherapy | | Fistula ^a^, constipation, defecation urgency ^a^, fecal incontinence |
| Verweij, 2023 | | 51 | | RT and surgery | | Clustering of stools ^a^, defecation urge ^a^, incontinence for flatus, defecation frequency ^a^, incontinence for liquid stools, diarrhea, fatigue, cystitis ^a^, dermatitis, urine incontinence |
| Wang, 2020 | | 3193 | | Surgery combined with other treatments | | Urinary retention ^a^ |
| Wang, 2022 | 62 | | CRT | | Proctitis ^a^, radiation-dermatitis, anal blood loss ^a^, urinary frequency, rectal pain ^a^, blood in stools ^a^, fecal incontinence, bowel movement frequency ^a^ | |
| Wee, 2018 | 859 | | Chemoradiation combined with other treatments | | Diarrhea, proctitis ^a^ | |
| Yamada, 2022 | 77 | | RT | | Skin reaction, pain, neuropathy | |
| Yamazaki, 2022 | 38 | | CRT and surgery or CRT and chemotherapy | | Dehydration ^a^, diarrhea, fatigue, rash, syncope ^a^ | |
| Zimmerman, 2022 | 34 | | Chemotherapy, CRT, and surgery | | Diarrhea, nausea, vomiting, cystitis ^a^, urinary urgency, proctitis ^a^ | |
| Symptoms mentioned in literature with a prevalence <5% and symptoms inappropriate for patient self-reporting (e.g., neutropenia) are not shown. Prevalence is calculated irrespective of symptom grade and received treatment. Reported symptoms in the studies by Koike (2017), Liu (2021), Tsukamoto (2017) and Zhou (2021) did not reach ≥5% prevalence, studies are therefore not shown in this table.  ^a^ Symptom is currently not part of the PRO-CTCAE  ^b^ The following non-PRO-CTCAE symptoms were frequently reported with a prevalence ≥5%: ‘proctitis’ (25% of publications), ‘cystitis’ (18% of publications), and ‘stomatitis’ (14% of publications). Common PRO-CTCAE symptoms of ‘proctitis’ include ‘diarrhea’ ‘constipation’, and ‘pain in the abdomen’. Common PRO-CTCAE symptoms of ‘cystitis’ include ‘frequent urination’, ‘urinary urgency’, ‘painful urination’, and ‘change in usual urine color’. Common PRO-CTCAE symptoms of ‘stomatitis’ include ‘mouth or throat sores’.  CRT, chemoradiation; RT, radiotherapy | | | | | | |

| **Online Resource 1.4. Mean relevance scores for each PRO-CTCAE symptom scored by HCPs and patients during the interview rounds.** | | |
| --- | --- | --- |
| PRO-CTCAE symptom | Mean relevance score ^a^ | |
|  | HCPs (n=15) | Patients (n=15) |
| Fatigue ^b^ | **3.67** | **2.27** |
| Pain during vaginal sex ^b, c^ | **3.27** | **2.00** |
| Bloating of the abdomen ^b^ | **2.60** | **2.00** |
| Delayed orgasm | **2.33** | 1.93 |
| Inability to reach orgasms | **2.87** | 1.87 |
| Ability to achieve and maintain erection ^b^ | **3.40** | 1.80 |
| Insomnia | **3.07** | 1.80 |
| Diarrhea ^b^ | **3.80** | 1.73 |
| Ejaculation problems | **3.20** | 1.67 |
| Decreased libido ^b^ | **3.13** | 1.67 |
| Frequent urination | **3.07** | 1.67 |
| Numbness and tingling | **2.67** | 1.67 |
| Decreased appetite | **3.20** | 1.60 |
| Sadness | **2.87** | 1.57 |
| Urinary urgency | **2.93** | 1.53 |
| Taste changes | **2.80** | 1.53 |
| Increased sweating | 1.47 | 1.53 |
| Constipation ^b^ | **3.33** | 1.47 |
| Pain in the abdomen ^b^ | **3.33** | 1.47 |
| Concentration | **3.07** | 1.47 |
| Flatulence/gas | **2.80** | 1.47 |
| Fecal incontinence ^b^ | **3.93** | 1.40 |
| General pain ^b^ | **3.53** | 1.40 |
| Nausea | **3.07** | 1.40 |
| Anxiety ^b^ | **3.07** | 1.40 |
| Skin dryness | **2.13** | 1.40 |
| Painful urination ^b^ | **3.00** | 1.33 |
| Feeling that nothing could cheer you up ^b^ | **2.53** | 1.33 |
| Itching skin | **2.00** | 1.33 |
| Mouth/throat sores | 1.73 | 1.33 |
| Difficulty swallowing | 1.00 | 1.33 |
| Wheezing | 1.00 | 1.33 |
| Hand-foot syndrome ^b^ | **2.93** | 1.27 |
| Muscle pain | 1.53 | 1.27 |
| Nail discoloration | 1.47 | 1.27 |
| Voice quality changes | 1.00 | 1.27 |
| Radiation skin reaction | **3.00** | 1.20 |
| Memory | **2.87** | 1.20 |
| Vomiting ^b^ | **2.73** | 1.20 |
| Rash | **2.33** | 1.20 |
| Dizziness | 1.80 | 1.20 |
| Change in usual urine color | 1.67 | 1.20 |
| Arthralgia/joint pain | 1.33 | 1.20 |
| Urinary incontinence ^b^ | **3.13** | 1.13 |
| Shortness of breath | 1.80 | 1.13 |
| Headache | 1.67 | 1.13 |
| Hiccups | 1.53 | 1.13 |
| Pain swelling at injection site | 1.47 | 1.13 |
| Cracking at the corners of the mouth (cheilosis/cheilitis) | 1.47 | 1.13 |
| Chills | 1.80 | 1.07 |
| Bedsores/pressure sores | 1.73 | 1.07 |
| Heartburn | 1.67 | 1.07 |
| Hair loss | 1.67 | 1.07 |
| Dry mouth | 1.60 | 1.07 |
| Swelling (arm/leg) | 1.40 | 1.07 |
| Cough | 1.33 | 1.07 |
| Breast swelling and tenderness | 1.33 | 1.07 |
| Hives | 1.20 | 1.07 |
| Blurred vision | 1.00 | 1.07 |
| Visual floaters | 1.00 | 1.07 |
| Vaginal dryness ^c^ | **2.93** | 1.00 |
| Vaginal discharge ^c^ | **2.27** | 1.00 |
| Darkening skin | **2.13** | 1.00 |
| Hot flashes | **2.00** | 1.00 |
| Nail loss | 1.93 | 1.00 |
| Irregular periods/vaginal bleeding ^c^ | 1.93 | 1.00 |
| Heart palpitations | 1.80 | 1.00 |
| Missed expected menstrual period ^c^ | 1.80 | 1.00 |
| Acne | 1.67 | 1.00 |
| Sensitivity to sunlight | 1.60 | 1.00 |
| Body odor | 1.47 | 1.00 |
| Ridged nails | 1.47 | 1.00 |
| Nosebleed | 1.40 | 1.00 |
| Bruising | 1.33 | 1.00 |
| Watery eyes | 1.27 | 1.00 |
| Ringing in ears | 1.13 | 1.00 |
| Stretch marks | 1.00 | 1.00 |
| Hoarseness | 1.00 | 1.00 |
| Light flashes | 1.00 | 1.00 |
| Decreased sweating | 1.00 | 1.00 |
| Mean relevance scores are ordered from high to low according to patient scores. Bold indicates a mean relevance score ≥2.00  ^a^ HCPs and patients completed the PRO-CTCAE questionnaire and scored each item for relevance on a scale from 1 (not relevant) to 4 (very relevant).  ^b^ Symptom included in final PRO-CTCAE subset  ^c^ Symptom only scored by n=3 female patients  HCP, health care provider; PRO-CTCAE, Patient Reported Outcome version of the Common Terminology Criteria for Adverse Events | | |

| **Online Resource 1.5. Mean relevance score and number of times a PRO-CTCAE symptom was mentioned as part of the top ten during the semi-structured interviews with health care providers, according to treatment** | | | | | | |
| --- | --- | --- | --- | --- | --- | --- |
| PRO-CTCAE  symptom | Involved in care concerning treatment modality ^a^ | | | | | |
|  | Radiotherapy (n=8) | | Surgery (n=6) | | Chemotherapy (n=3) | |
|  | Mean relevance score | times in top 10 | Mean relevance score | times in top 10 | Mean relevance score | times in top 10 |
| *Gastrointestinal disorders* | | | | | | |
| Pain in the abdomen ^b^ | **3.63** | 2 | **3.00** | 1 | **3.00** | 0 |
| Bloating of the abdomen ^b^ | **3.00** | 3 | 2.00 | 0 | 2.33 | 0 |
| Constipation ^b^ | **3.63** | 3 | **3.17** | 2 | 2.67 | 0 |
| Diarrhea ^b^ | **3.88** | 5 | **3.67** | 6 | **3.33** | 3 |
| Taste changes | **3.25** | 1 | 2.33 | 1 | **3.33** | 1 |
| Decreased appetite | **3.38** | 2 | **3.17** | 3 | **3.33** | 1 |
| Fecal incontinence ^b^ | **4.00** | 7 | **4.00** | 4 | **3.67** | 2 |
| Flatulence | **3.13** | 2 | 2.50 | 0 | 2.00 | 0 |
| Nausea | **3.25** | 1 | **3.00** | 1 | **3.33** | 2 |
| Vomiting ^b^ | 2.75 | 0 | **3.00** | 0 | **3.00** | 1 |
| *Reproductive and sexual disorders* | | | | | | |
| Pain during vaginal sex ^b^ | **3.38** | 3 | **3.33** | 2 | **2.67** | 0 |
| Ability to achieve and maintain erection ^b^ | **3.50** | 4 | **3.67** | 6 | **3.00** | 1 |
| Decreased libido ^b^ | **3.38** | 2 | **3.17** | 1 | **3.00** | 0 |
| Inability to reach orgasms | 2.75 | 1 | **3.17** | 0 | 2.67 | 0 |
| Ejaculation | **3.38** | 0 | **3.33** | 2 | **3.00** | 0 |
| Vaginal dryness | **3.00** | 3 | **3.00** | 4 | 2.67 | 1 |
| *Renal and urinary disorders* | | | | | | |
| Frequent urination | **3.50** | 3 | 2.83 | 1 | 2.33 | 0 |
| Urinary incontinence ^b^ | **3.25** | 2 | **3.33** | 3 | 2.33 | 1 |
| Painful urination ^b^ | **3.50** | 4 | 2.50 | 1 | 2.67 | 1 |
| Urinary urgency | **3.38** | 3 | 2.67 | 1 | 2.33 | 0 |
| *Sleep/wake and mood* | | | | | | |
| Fatigue ^b^ | **3.50** | 7 | **3.83** | 5 | **4.00** | 2 |
| Anxiety ^b^ | **3.00** | 2 | **3.33** | 1 | 2.67 | 2 |
| *Neurological disorders, memory, and concentration* | | | | | | |
| Numbness and tingling | **3.13** | 1 | 2.33 | 0 | **3.00** | 1 |
| Concentration | 2.88 | 1 | **3.33** | 2 | **3.67** | 3 |
| *Pain, mouth, dermatological, and respiratory disorders* | | | | | | |
| General pain ^b^ | **3.50** | 2 | **3.50** | 3 | **4.00** | 1 |
| Mouth/throat sores | 2.00 | 0 | 1.50 | 0 | 2.33 | 1 |
| Hand-foot syndrome ^b^ | **3.38** | 4 | 2.50 | 2 | **3.33** | 3 |
| Radiation skin reaction | **3.63** | 5 | 2.00 | 0 | **3.33** | 1 |
| Only symptoms that were mentioned at least once as part of the ten symptoms that, based on HCPs professional experience, had the greatest impact on rectal cancer patients and should thus be included in the final PRO-CTCAE subset, are shown. Bold indicates a mean relevance score ≥3.00. HCPs completed the PRO-CTCAE questionnaire and scored each item for relevance on a scale from 1 (not relevant) to 4 (very relevant) and were asked to identify a subset of ten symptoms that, based on their professional experience, had the greatest impact on rectal cancer patients and should thus be included in the final PRO-CTCAE subset.  ^a^ The HCP sample consisted of seven (47%) radiation oncologists, four (27%) surgeons, three (20%) medical oncologists, and one (7%) nurse specialist.  ^b^ Symptom included in final PRO-CTCAE subset  PRO-CTCAE, Patient Reported Outcome version of the Common Terminology Criteria for Adverse Events; HCP, health care provider; HRQOL, health-related quality of life. | | | | | | |
