# Supplementary material for: Using a modified Delphi procedure to select a PRO-CTCAE-based subset for patient-reported symptomatic toxicity monitoring in rectal cancer patients
Source: Qual Life Res. 2024 Sep 8;33(11):3013–26. doi: 10.1007/s11136-024-03767-0 (PMC11541275; doi:10.1007/s11136-024-03767-0)
Supplement: Supplementary file 2 — Supplementary file2 (DOCX 28 KB) [file 11136_2024_3767_MOESM2_ESM.docx]

**Online Resources 2**

Article title: *Using a modified Delphi procedure to select a PRO-CTCAE-based subset for patient-reported symptomatic toxicity monitoring in rectal cancer patients.*

Journal: *Quality of Life Research*

Authors: *Yvonne M. Geurts, Femke Peters, Esther Feldman, Jeanine Roodhart, Milan Richir, Jan Willem T. Dekker, Geerard Beets, Jeltsje S. Cnossen, Patricia Bottenberg, Martijn Intven, Marcel Verheij, Kelly M. de Ligt, Iris Walraven.*

Corresponding author: *Yvonne M. Geurts, Department of IQ Health, Radboud university medical center, The Netherlands,* [*yvonne.m.geurts@radboudumc.nl*](mailto:yvonne.m.geurts@radboudumc.nl)

**NCI-PRO-CTCAE® CUSTOM SURVEY
Item subset derived from PRO-CTCAE® Item Library Version 1.0**

**https://healthcaredelivery.cancer.gov/pro-ctcae/builder.html**

**As individuals go through treatment for their cancer they sometimes experience different symptoms and side effects. For each question, please select the one response that best describes your experiences over the past 7 days…**

| **1a.** In the last 7 days, how OFTEN did you have VOMITING? | | | | |
| --- | --- | --- | --- | --- |
| Ο Never | Ο Rarely | Ο Occasionally | Ο Frequently | Ο Almost constantly |
| **1b.** In the last 7 days, what was the SEVERITY of your VOMITING at its WORST? | | | | |
| Ο None | Ο Mild | Ο Moderate | Ο Severe | Ο Very severe |

| **2a.** In the last 7 days, how OFTEN did you have BLOATING OF THE ABDOMEN (BELLY)? | | | | |
| --- | --- | --- | --- | --- |
| Ο Never | Ο Rarely | Ο Occasionally | Ο Frequently | Ο Almost constantly |
| **2b.** In the last 7 days, what was the SEVERITY of your BLOATING OF THE ABDOMEN (BELLY) at its WORST? | | | | |
| Ο None | Ο Mild | Ο Moderate | Ο Severe | Ο Very severe |

| **3a.** In the last 7 days, what was the SEVERITY of your CONSTIPATION at its WORST? | | | | |
| --- | --- | --- | --- | --- |
| Ο None | Ο Mild | Ο Moderate | Ο Severe | Ο Very severe |

| **4a.** In the last 7 days, how OFTEN did you have LOOSE OR WATERY STOOLS (DIARRHEA/DIARRHOEA)? | | | | |
| --- | --- | --- | --- | --- |
| Ο Never | Ο Rarely | Ο Occasionally | Ο Frequently | Ο Almost constantly |

| **5a.** In the last 7 days, how OFTEN did you have PAIN IN THE ABDOMEN (BELLY AREA)? | | | | |
| --- | --- | --- | --- | --- |
| Ο Never | Ο Rarely | Ο Occasionally | Ο Frequently | Ο Almost constantly |
| **5b.** In the last 7 days, what was the SEVERITY of your PAIN IN THE ABDOMEN (BELLY AREA) at its WORST? | | | | |
| Ο None | Ο Mild | Ο Moderate | Ο Severe | Ο Very severe |
| **5c.** In the last 7 days, how much did PAIN IN THE ABDOMEN (BELLY AREA) INTERFERE with your usual or daily activities? | | | | |
| Ο Not at all | Ο A little bit | Ο Somewhat | Ο Quite a bit | Ο Very much |

| **6a.** In the last 7 days, how OFTEN did you LOSE CONTROL OF BOWEL MOVEMENTS? | | | | |
| --- | --- | --- | --- | --- |
| Ο Never | Ο Rarely | Ο Occasionally | Ο Frequently | Ο Almost constantly |
| **6b.** In the last 7 days, how much did LOSS OF CONTROL OF BOWEL MOVEMENTS INTERFERE with your usual or daily activities? | | | | |
| Ο Not at all | Ο A little bit | Ο Somewhat | Ο Quite a bit | Ο Very much |

| **7a.** In the last 7 days, what was the SEVERITY of your HAND-FOOT SYNDROME (A RASH OF THE HANDS OR FEET THAT CAN CAUSE CRACKING, PEELING, REDNESS OR PAIN) at its WORST? | | | | |
| --- | --- | --- | --- | --- |
| Ο None | Ο Mild | Ο Moderate | Ο Severe | Ο Very severe |

| **8a.** In the last 7 days, how OFTEN did you have PAIN? | | | | |
| --- | --- | --- | --- | --- |
| Ο Never | Ο Rarely | Ο Occasionally | Ο Frequently | Ο Almost constantly |
| **8b.** In the last 7 days, what was the SEVERITY of your PAIN at its WORST? | | | | |
| Ο None | Ο Mild | Ο Moderate | Ο Severe | Ο Very severe |
| **8c.** In the last 7 days, how much did PAIN INTERFERE with your usual or daily activities? | | | | |
| Ο Not at all | Ο A little bit | Ο Somewhat | Ο Quite a bit | Ο Very much |

| **9a.** In the last 7 days, what was the SEVERITY of your FATIGUE, TIREDNESS, OR LACK OF ENERGY at its WORST? | | | | |
| --- | --- | --- | --- | --- |
| Ο None | Ο Mild | Ο Moderate | Ο Severe | Ο Very severe |
| **9b.** In the last 7 days, how much did FATIGUE, TIREDNESS, OR LACK OF ENERGY INTERFERE with your usual or daily activities? | | | | |
| Ο Not at all | Ο A little bit | Ο Somewhat | Ο Quite a bit | Ο Very much |

| **10a.** In the last 7 days, how OFTEN did you feel ANXIETY? | | | | |
| --- | --- | --- | --- | --- |
| Ο Never | Ο Rarely | Ο Occasionally | Ο Frequently | Ο Almost constantly |
| **10b.** In the last 7 days, what was the SEVERITY of your ANXIETY at its WORST? | | | | |
| Ο None | Ο Mild | Ο Moderate | Ο Severe | Ο Very severe |
| **10c.** In the last 7 days, how much did ANXIETY INTERFERE with your usual or daily activities? | | | | |
| Ο Not at all | Ο A little bit | Ο Somewhat | Ο Quite a bit | Ο Very much |

| **11a.** In the last 7 days, how OFTEN did you FEEL THAT NOTHING COULD CHEER YOU UP? | | | | |
| --- | --- | --- | --- | --- |
| Ο Never | Ο Rarely | Ο Occasionally | Ο Frequently | Ο Almost constantly |
| **11b.** In the last 7 days, what was the SEVERITY of your FEELINGS THAT NOTHING COULD CHEER YOU UP at their WORST? | | | | |
| Ο None | Ο Mild | Ο Moderate | Ο Severe | Ο Very severe |
| **11c.** In the last 7 days, how much did FEELING THAT NOTHING COULD CHEER YOU UP INTERFERE with your usual or daily activities? | | | | |
| Ο Not at all | Ο A little bit | Ο Somewhat | Ο Quite a bit | Ο Very much |

| **12a.** In the last 7 days, what was the SEVERITY of your PAIN OR BURNING WITH URINATION at its WORST? | | | | |
| --- | --- | --- | --- | --- |
| Ο None | Ο Mild | Ο Moderate | Ο Severe | Ο Very severe |

| **13a.** In the last 7 days, how OFTEN did you have LOSS OF CONTROL OF URINE (LEAKAGE)? | | | | |
| --- | --- | --- | --- | --- |
| Ο Never | Ο Rarely | Ο Occasionally | Ο Frequently | Ο Almost constantly |
| **13b.** In the last 7 days, how much did LOSS OF CONTROL OF URINE (LEAKAGE) INTERFERE with your usual or daily activities? | | | | |
| Ο Not at all | Ο A little bit | Ο Somewhat | Ο Quite a bit | Ο Very much |

| **14a.** In the last 7 days, what was the SEVERITY of your DIFFICULTY GETTING OR KEEPING AN ERECTION at its WORST? | | | | | | |
| --- | --- | --- | --- | --- | --- | --- |
| Ο None | Ο Mild | Ο Moderate | Ο Severe | Ο Very severe | Ο Not sexually active | Ο Prefer not to answer |

| **15a.**  In the last 7 days, what was the SEVERITY of your DECREASED SEXUAL INTEREST at its WORST? | | | | | | |
| --- | --- | --- | --- | --- | --- | --- |
| Ο None | Ο Mild | Ο Moderate | Ο Severe | Ο Very severe | Ο Not sexually active | Ο Prefer not to answer |

| **16a.** In the last 7 days, what was the SEVERITY of your PAIN DURING VAGINAL SEX at its WORST? | | | | | | |
| --- | --- | --- | --- | --- | --- | --- |
| Ο None | Ο Mild | Ο Moderate | Ο Severe | Ο Very severe | Ο Not sexually active | Ο Prefer not to answer |

| **OTHER SYMPTOMS** | | | | | | |
| --- | --- | --- | --- | --- | --- | --- |
| Do you have any other symptoms that you wish to report? | | | | | | |
| Ο Yes | | | Ο No | | | |
| **Please list any other symptoms:** | | | | | | |
| 1. | In the last 7 days, what was the SEVERITY of this symptom at its WORST? | | | | | |
|  | O None | O Mild | | O Moderate | O Severe | O Very Severe |
| 2. | In the last 7 days, what was the SEVERITY of this symptom at its WORST? | | | | | |
|  | O None | O Mild | | O Moderate | O Severe | O Very Severe |
| 3. | In the last 7 days, what was the SEVERITY of this symptom at its WORST? | | | | | |
|  | O None | O Mild | | O Moderate | O Severe | O Very Severe |
| 4. | In the last 7 days, what was the SEVERITY of this symptom at its WORST? | | | | | |
|  | O None | O Mild | | O Moderate | O Severe | O Very Severe |
| 5. | In the last 7 days, what was the SEVERITY of this symptom at its WORST? | | | | | |
|  | O None | O Mild | | O Moderate | O Severe | O Very Severe |
